# The Work of London Hospitals and Infirmaries. West End Hospital: News from the Staff in Belgium—A London Secretary as Artillery Officer—New Headquarters of British Red Cross Society—Homerton and Bow Infirmaries

**Published:** 1914-09-26

**Authors:** 


### HOSPITALS AND THE WAR.

(From our Special Correspondents.)

### The Work of London Hospitals and Infirmaries.

### WEST END HOSPITAL: NEWS FROM THE STAFF IN BELGIUM.

A number of members of the committee and a considerable proportion of the medical staff of the West End Hospital for Nervous Diseases are serving with the fighting or medical forces of the Empire.

The chairman, Lord Hindlip, has taken up the duties of a King's Messenger; the treasurer, Colonel Willoughby, commands a brigade of Territorials; the Earl of Dunmore, V.C., and Captain Harry Graham rejoin their regiments. Of the medical staff, Dr. Macnamara was called up by the Admiralty on the outbreak of hostilities, and Dr. Carlill and Dr. Phillips (anæsthetist) have been granted temporary appointments in the Navy. Dr. Golla has a field ambulance, and Dr. Vincent Denne (dental surgeon) is serving with the City of London Territorials. Mr. Ballance and Dr. Purves Stewart are attached to base hospitals, but are still in attendance at the hospital. The masseur left early for Belgium in the Royal Army Medical Corps.

Miss Thorpe, the matron, left London to serve under the Belgian Red Cross flag, and after leaving Brussels was two days at Tirlemont and then two days at Perk in a German field hospital. She was last heard of from Mons, where her party had set up forty-four beds in a house, and were very busy. The work at the hospital has been taken up by Miss Helen Stuart-Smith, the senior sister. Notwithstanding the loss of so many of its officers, the work of the hospital is in full swing. Beds have been offered and accepted by the War Office, and demonstrations were given by some of the sisters to large numbers of Mr. Cantlie's Red Cross pupils.

### A LONDON SECRETARY AS ARTILLERY OFFICER.

To those hospital secretaries already noted in The Hospital as being on active service, our readers will be interested to hear that the name of Mr. Edward A. Attwood, secretary of the London Homœopathic Hospital, must be added. He has been transferred from the Territorial Field Artillery to the Regular Army, and has accepted a commission as captain in the Royal Field Artillery for the duration of the war.

We understand that Mr. W. Hammond, the senior clerk and steward, will undertake the duties of secretary during Captain Attwood's absence.

### NEW HEADQUARTERS OF BRITISH RED CROSS SOCIETY.

The British Red Cross Society asks us to state that on Monday last, the 21st instant, its London headquarters were removed to 83 Pall Mall, where spacious accommodation has been placed at the Society's disposal by the generosity of the committee of the Royal Automobile Club, the owners of the building. For the information of those who have occasion to call it may be added that the offices will be found next door to the Automobile Club.

In leaving Devonshire House, which the Duke and Duchess of Devonshire on the outbreak of war generously placed at the disposal of the Society for an indefinite period, the committee of the British Red Cross Society has expressed its grateful thanks to the Duke and Duchess for their great kindness. It is solely the result of increasing pressure of business and its corresponding needs which has made it necessary to remove to offices which structurally provide more suitable accommodation. Subscriptions should now be addressed to Lord Rothschild at 83 Pall Mall, to which address any other correspondence relating to the British Red Cross Society should henceforward be sent.

We are asked to state that the prevalent reports that the British Red Cross Society has a sufficient supply of garments for the sick and wounded are quite incorrect. So recently as Sunday last the following telegram was received from Rouen at 83 Pall Mall, the headquarters of the Society, from Sir Alfred Keogh, the Society's Chief Commissioner in France:—"Send with utmost speed to Rouen, in addition to articles now on way shirts, pyjamas, sheets, pillow-slips, mackintosh sheets, slippers, and socks. Send thousands of above. You cannot send too many.—Keogh." All articles should be addressed to British Red Cross Society, Stores Department, 83 Pall Mall, London, S.W.

### HOMERTON AND BOW INFIRMARIES.

The City of London Board of Guardians, in addition to placing at the disposal of the War Office vacant beds in their infirmary at Homerton, as recorded in the last issue of The Hospital, p. 667, have also rendered additional services to the authorities. They have placed the south block at Bow Infirmary, with accommodation for 200 wounded soldiers, at the disposal of the War Office; expressed readiness to receive Belgian refugees at Thavies Inn and Homerton; offered the services of a large number of skilled clerks to ease the burden at the recruiting offices between the hours of 3 and 10 p.m.; placed the services of the medical staff at the disposal of the authorities for the examination of recruits; sworn in a number of the staff as special constables for the protection of the offices; sent twenty-five members of the staff to the Front, and received upwards of fifty prisoners of war. This is an example of patriotism which might well be considered by other Boards of Guardians with plenty of spare men and accommodation.

